# *Maskne*: The Epidemic within the Pandemic: From Diagnosis to Therapy

**DOI:** 10.3390/jcm11030618

**Published:** 2022-01-26

**Authors:** Cristina Beatrice Spigariolo, Serena Giacalone, Gianluca Nazzaro

**Affiliations:** 1 Dermatology Unit, Fondazione IRCCS Ca’ Granda Ospedale Maggiore Policlinico, 20122 Milan, Italy; cristina.spigariolo@gmail.com (C.B.S.); serenagiacalone92@gmail.com (S.G.); 2 Department of Pathophysiology and Transplantation, Università degli Studi di Milano, Via Pace 9, 20122 Milan, Italy

**Keywords:** *maskne*, acne, mask, pandemic, SARS-CoV-2, COVID-19, facial dermatosis

## Abstract

*Maskne*, a newly coined term deriving from the contraction of mask-related acne, is a form of mechanical acne resulting from continuous textile–skin adherence and friction. Prolonged mask use heats up the face environment, thus modifying skin microbiota and sebum production. Although effective prevalence is unknown, since the pandemic began and the prolonged use of masks was extended to the general population, *maskne* has been a frequent topic of consultation among dermatologists. This term has been successfully introduced into common language, with more than 200,000 hashtags on social media, where it is also possible to find “home remedies” that can worsen this dermatosis. The aim of this paper is to discuss the pathogenesis, address clinicians on the correct differential diagnoses among facial dermatoses, and move towards the correct therapy.

## 1. Introduction

In last two years of the SARS-CoV-2 outbreak, people have had to modify their daily routine, introducing new habits and devices to reduce the risk of infection. Among all personal protective equipment (PPE), facial masks are the most employed and effective instrument to maintain a state of health [1]. Their use, first only among healthcare workers and then among general population, has led to an increase of facial dermatoses, i.e., acne, rosacea, seborrheic dermatitis, and contact dermatitis. This phenomenon was so prevalent that a new description term, *maskne*, has been coined. *Maskne* is a contraction of mask-related acne and describes a form of acne in the O-area secondary to the prolonged use of facial masks [2]. Although effective prevalence is unknown, there is currently an increasing request for consultations due to acne onset or its worsening [3].

In a Turkish survey, conducted from December 2020 to February 2021, 101 healthcare workers (HCW) were screened for facial dermatoses and it emerged that acne prevalence was present in 55.4% of participants [4]. Similar findings resulted from another survey among HCW from Pakistan [5]. Acneiform eruptions have been documented in two larger cohorts of 454 [6] and 400 [7] subjects, where prevalence was respectively assessed to 39.9% and 43% of all adverse skin reactions due to prolonged face mask use.

Additional studies confirmed that rosacea, irritant contact dermatitis, and acne [8,9] worsened after mask use, even among children [10]. It is also acknowledged that acne could contribute to mask-induced itch [11].

Moreover, some risk factors, such as greater stress, the sleep deprivation HCW undergo during their shifts in COVID wards, and the consequent increasing in cortisol secretion may contribute to the development of acne [12].

Nevertheless, mask-related acne was also reported in a Chinese survey conducted in 2006 during in 2006 during the SARS epidemic, showing prevalence of acne in 59.6% of 109 workers with facial dermatoses [13].

## 2. Methods

This review was conducted by searching the terms “*maskne*”, “mask related acne”, and “facial dermatosis and mask” on PubMed and Embase. Articles containing “*maskne*” and/or “mask related acne” in their titles and articles with “facial dermatosis and mask” in their titles dealing with acne were included and analyzed. Clinical trials, reviews, case series, and case reports were included. Facial dermatosis related to oxygen masks was not considered. The search was limited to article written in English.

## 3. Definition and Pathogenesis

*Maskne* is a form of mechanical acne [6,14]. resulting from continuous textile–skin adherence and friction [15]. Furthermore, prolonged mask use determines a hot-humid microclimate on the skin surface, thus modifying sebum production and consequently microbiota [16]. The term *maskne* refers both to new diagnoses and to aggravation of pre-existing acneiform eruption [17].

### 3.1. The Role of Microbiota

Mask use is responsible of the disruption of skin microbiota balance causing bacterial dysbiosis. It has been demonstrated that cutaneous microbiota develops with the age [18]: infant skin is more sensitive to inflammatory diseases such as atopic dermatitis and diaper dermatitis and to infections such as *S. Aureus* and candidiasis. Maturation of microbiota from childhood to adulthood has been demonstrated to have a pivotal role in preventing inflammatory skin diseases. During the transition through puberty, sebum overproduction has been linked to colonization by *Cutibacterium acnes*, while its decrease seems to be related with the lower sebum secretion observed in aging skin.

The main actor in acne is *Cutibacterium acnes*, which, as the most prevalent species, accounts for 90% of microbiota of the pilo-sebaceous unit. Its density varies with age, increasing from adolescence to middle adult age and then decreasing from 40–50 years old. *C. acnes* is involved in a double mechanism: on one hand, colonies of *C. acnes* use sebum lipids as a metabolic intermediate to promote their growth, on the other, they favor sebum production by increasing the activity of diacylglycerol acyltransferase. Moreover, the porphyrins released by *C. acnes* are catalytic factors for the oxidation of squalene, a main component of sebum [19]. Dysbiosis seems to select pathogen species of *C. acnes* and activates innate immunity causing cutaneous inflammation [20]. A recent study [21] suggests that the severity of inflammation in acne may be explained by the loss of diversity of *C. acnes* phylotypes with the selection of phylotype IA1, which enhances the innate immune system, thereby promoting the release of inflammatory cytokines.

In any case, if correctly balanced, *C. acnes* is considered a fundamental commensal for skin health because of its role in maintaining low pH, thus preventing *Staphylococcus aureus* and *Streptococcus* spp. proliferation.

The significance of Malassezia in acne is not totally clear, though it is known for its pathogenetic role in seborrheic dermatitis and in *Pityrosporum folliculitis*, a clinical mimic of acne. Malassezia hydrolyzes free fatty acids in sebum, which may affect the abnormal keratinization of hair follicles and promote the secretion of pro-inflammatory cytokines from keratinocytes and monocytes [19].

Dysbiosis is also responsible of other skin diseases; in particular, flare-ups of eczema are significantly related to *S. Aureus* colonization, Gram-negative folliculitis may aggravate acne, Fusobacteria are involved in perioral dermatitis, and *Demodex follicolorum* seems to be linked to rosacea [19].

### 3.2. The Microenvironment: Temperature and pH

Different studies have reported the aggravation of acne during the summer season in relation to higher temperature. In particular, the most severe cases have been observed in tropical and subtropical climates, likely due to humidity [15,18].

The close correlation between high temperature and acne flare can be explained by the effect of higher temperature on the sebum excretion rate. Sebum excretion increases by 10% for each 1 °C rise. Furthermore, squalene could become significantly greater in surface lipids when temperature increases [19]. Moreover, the increase of humidity plays a role through the poral occlusive effect, irritation, and swelling of the skin. Both sweat and increased humidity may cause acute obstruction and aggravate acne [20].

The same alterations are reproduced by the facial mask use because:

- It makes the air temperature between skin and mask higher due to the restricted area and its closes adherence to the skin, particularly at the boundaries;
- It increases sweat retention, especially in people affected by hyperhidrosis;
- It reduces air recirculation, favoring the deposition of exhaled damp hair and toxins.

Therefore, masks may produce a microclimate similar to a greenhouse, thus favoring microbiota that contribute to the development of acne.

A study conducted on 20 participants aimed to compare facial skin temperature and heat flow using medical-surgical equipment [22]. A statistically significant difference in humidity, heat, breathing difficulty, and discomfort was present. Infrared thermography images demonstrated temperature changes at the perioral region and superior lip immediately after removal of the mask, compared with baseline conditions in both types of PPE, while no temperature augmentations were observed on the forehead, cheeks, and nose/mouth.

As far as the pH of skin, there is no consensus if lower pH contributes the development of acne [23]; However, changes in pH contribute to dysbiosis and thus promote the development of this disease.

Finally, mask-wearing might create a new intertriginous area where different type of microorganisms can grow [13,20]

### 3.3. Characteristics of Masks

FFP2/KN95 masks are greater risk factors for the development of acne than surgical masks because of their higher humidity, occlusion, and temperature [4].

In the study of Techasatian et al. [6], it emerged that four types of masks are frequently used by the general population: surgical masks, cloth masks, surgical masks covered by a piece of cloth, and N95/FFP2 mask. This paper [6] showed that different factors are responsible for skin side effects. The first one is the mask type: surgical masks and surgical masks covered by a piece of cloth are related to major risks; immediately followed by the duration of mask wearing (especially over 4–6 h/day) and reutilization of the same mask. In addition, the use of sanitizers for masks seemed to have a dual effect: direct skin irritation and predisposition to the occlusion mechanism.

This discrepancy between the causative role of surgical masks and KN95/FFP2 might be explained by the fact that KN95 are more utilized by healthcare workers and less among the general population.

Cloth masks were more involved than surgical and FFP2/KN95 masks in the development of acne in an Indian study [24]. The explanation given by the authors is that people used cloth masks for many days without adequate washing and hygiene practices, with a consequent accumulation of sweat and environment dirt.

It has been also demonstrated that textile dyes, rubber, rubber antioxidants, chemical adhesives, and formaldehyde may influence the development of allergic (ACD) and irritant contact dermatitis (ICD) over areas in contact with face masks [25,26].

## 4. Clinical Features of *Maskne*

Acne related to masks occurred more frequently on the chin than the cheeks and it appeared as mild papular eruptions, accompanied by comedones and seborrhea [1,4].

## 5. Diagnostic Criteria

We propose five diagnostic criteria for *maskne*:

- Appearance of acne after six weeks of mask-use or aggravation of pre-existing acne in the mask area [15,16];
- Elementary lesions as papules, pustules, and comedones;
- Localization in the area of mask or O-area;
- Temporal relationship with mask use: aggravation/development of acne with prolonged usage (>4–6 h/day [6]) and improvement when not worn for a long period;
- Exclusion of other dermatoses such as perioral dermatitis, rosacea, seborrheic dermatitis, ICD, and ACD [15]

Given the similarities between acne related to mask and acne vulgaris, *maskne* classification may reflect the one adopted for acne according to European Guidelines [27].

## 6. Other Mask Related Facial Dermatosis: Differential Diagnosis

*Maskne* is only one of the multiple PPE-related facial dermatoses that have been reported since the SARS-CoV-2 outbreak began [16]. Before starting treatment, it is of high importance to consider all possible differential diagnosis. Below, we have listed the most common causes of dermatological eruption triggered or exacerbated by using facial masks.

### 6.1. Irritant Contact Dermatitis

Irritant contact dermatitis (ICD) is an eczematous eruption resulting from direct contact with chemicals or physical irritants. Clinically, lesions begin with erythema, oedema, and vesicles limited to the contact area, then scaling, lichenification, erosions, and ulcerations can appear. The patient may complain of burning and itching sensations. Risk factors include a personal or familial history of atopic dermatitis; severity varies depending on the type of irritant and time of exposure. Specifically, mask-related ICD involves pressure areas (forehead, cheeks, nasal bridge, and ears) in prolonged PPE users (mainly more than 6 h/day). Eruption tends to ameliorate with regular mask breaks and application of moisturizers [16]. To reduce pressure in the convex areas of the face, some authors have suggested the possibility of dressing mask margins with silicon [28].

### 6.2. Allergic Contact Dermatitis

Allergic contact dermatitis (ACD) is an exogenous eczema induced by a delayed IV hypersensitivity reaction to an external allergen. Since clinical manifestations can mimic ICD, patch test positivity is often an essential instrument to diagnosis. Like mask ICD, the severity of facial ACD is proportional to PPE time of exposure and resolution can be achieved only after complete avoidance of causative allergens [16]. Exposure of the following substances may be responsible for ACD [29]:

- Metal wires (nickel and cobalt) are present to adapt masks to the facial convex area. Metals ions can reach the skin when masks are consumed or repetitively used;
- Formaldehyde is present in raw materials as a contaminant released from product packaging and a by-product of polypropylene degradation;
- Adhesive chemicals such as methyldibromo glutaronitrile are present in masks;
- Rubber accelerators are involved in the production of mask elastic bands and include substances such as thiurams, carbamates, dialkyl thioureas, and *N*-isopropyl-*N*-phenyl-p-phenylendiamine.

### 6.3. Rosacea

Rosacea is a common chronic inflammatory skin disease with different clinical subtypes. Since the presence of papules and pustules can mimic acne, evidence of multiple telangiectasias and absence of comedones are key signs to differentiate the two entities. Rosacea usually involves the “T zone” of the face, including convex areas such as forehead, nose, cheeks, and chin, beyond the area covered by masks. Since the outbreak began, rosacea-like eruptions limited to mask area and flares of chronic cases of rosacea have both been reported [16,30]. Similar to acne, in both cases etiopathogenetic mechanisms involve plural factors such as increased humidity, temperature changes, alteration of innate immunity, and microbiome dysbiosis [30].

### 6.4. Seborrheic Dermatitis

Seborrheic dermatitis (SD) is a very common skin disorder that affect all ages; predominantly, it has a bimodal distribution with a peak during infancy and adulthood and higher incidence among immunocompromised patients. It is characterized by greasy yellow scales overlying well defined erythematous patches. A key element to diagnosis is the typical facial distribution on nasolabial folds, eyebrows, ears, retroarticular folds, and scalp; extra-facial involvement includes the chest, axilla, and groin [31]. In the last two years, with the increasing use of facial mask, multiple studies have pointed out clinical worsening of patients with chronic SD. The increase of temperature created by the mask induces an increased sebum excretion rate, more on less equal to 10% for each 1 °C. These factors together may be responsible for higher permeability of the skin barrier, increasing sweating and abnormalities in *Malassezia* spp. proliferation [32].

### 6.5. Perioral Dermatitis

Perioral dermatitis (PD) is a benign eruption consisting of small inflammatory papules and pustules around the mouth [33]. Since the perioral region is the most common area of distribution, differentiate PD from *maskne* can be a diagnostic challenge. History of direct or indirect topical steroid and cosmetic usage and sparing of the vermillion border are characteristic of PD. However, long duration mask use can predispose or aggravated PD with similar mechanism to ACD, ICD, and SD [16].

### 6.6. Urticaria

Among various PPE complications, pressure and contact urticaria need to be mentioned [34]. In the first case, the immediate or delayed appearance of wheals is possible on sites of pressure. In the second case, urticaria can be elicited from mask allergens (e.g., formaldehyde); it typically appears immediately after contact and resolves within 24 h after avoidance of the trigger. While pressure urticaria involves specific area such as the nasal bridge, forehead, and cheeks, contact urticaria can appear all over the face [35].

### 6.7. Folliculitis

Facial folliculitis significantly resembles acne vulgaris, steroid acne, or *maskne*. Regardless of different aetiologies, it clinically appears with monomorphic papules and pustules, and rarely with nodules. Typically, it involves facial hair and is found mainly in men. Cutaneous swabs are recommended to exclude bacterial and fungal infection [16].

## 7. Advice and Therapies

Counseling about educational therapy, treatment expectations, and skin care is important for maximizing patients’ adherence to treatment.

The first approach consists in educational therapy. Suggested behaviors are as follows:

- Do not re-use the same mask for many days, according to the producer’s instructions [24];
- Avoid the use of sanitizers for mask [4];
- Wash hands before putting on the mask and after removing it [4];
- Apply non-comedogenic moisturizers before and after mask use [15];
- Replace FFP2/N95 and surgical masks after 3 days and 4 h, respectively [4,16];
- Take breaks of 15 min every 2 h from mask-use if feasible, always according to regulations and laws in force [4].

Generally, we do not recommend a specific type of mask, since masks must be chosen in relation to the personal risk of exposure. However, white color equipment reduces the possible risk of irritating/allergic contact dermatitis.

Since approved guidelines for treatment of *maskne* do not exist, instructions are taken from acne vulgaris therapies [15], as we do in our experience. The choice depends on the morphology of acne lesions (papules, pustules, comedones) and acne severity (mild, moderate, severe). As general rules, mild acne should be treated with topical products; a combination of topical agents or an association between topical and systemic therapies are suggested for moderate acne, while severe forms usually require both systemic and topical therapies [36]. In all cases, dermo-cosmetic products for daily skin care are highly recommended due to their synergistic effect with pharmacological agents and their role in maintenance therapy and management of side effects.

Herein, we propose a therapeutical scheme for *maskne* adapted from acne therapy guidelines compared with what is mentioned in literature about *maskne* and to our personal experience.

### 7.1. Topical Treatment of Maskne

Dermatologists may suggest a cleanser for acne-prone skin that removes sebum, dirt, cosmetics, and bacteria. In particular, gentle non-comedogenic antibacterial detergent with a pH between 5 and 7.3, close to normal skin, should be preferred [37]. Despite the common perception of an excess of seborrhea on the face, excessive face cleaning is not recommended, at most twice a day, in order to prevent inflammation and peeling and to avoid the rebound effect with consequent hypersecretion of sebum.

The application of dermo-cosmetic cream has the aim of sealing, moistening, and moisturizing the epidermis by reducing water loss, attracting water to the dermis. and makes the skin smooth and soft, respectively. It is also important to avoid scrubbing of affected areas and popping pimples to prevent irritation and increased inflammation [38]. The selection of dermo-cosmetic cream must be accurate. For example, natural moisturizing factors as sodium hyaluronate and polyglutamic acid are humectants that reduce transepidermal water loss without any irritation when worn under occlusion [20]. In contrast, some emollients (e.g., lanolin, glycerol stearate, glyceryl stearate, soy sterols, petrolatum, mineral oil, and dimethicone) may enhance their occlusive power under the mask. Softening creams containing niacinamide have sebostatic and anti-inflammatory effects [39]. Patients affected by hyperhidrosis could benefit from powder formulations that prevent occlusion and absorb excess moisture. Products based on zinc oxide formulations reduce humidity and are stable in powder compounds [20].

The most commonly used topical agents have been collected in Table 1. These molecules should be applied once a day for 4–12 weeks, preferably on evening [36,40].

According to Han et al. [24], patients suffering from *maskne* may benefit from the use of dermo-cosmetics for acne-prone skin. In *maskne,* antibiotics (AB) and fixed combinations of retinoids and AB, especially in hydrogel formulation, were more effective and safer [20] than agents such as benzoyl peroxide (PB), salicylic acid, and retinoids alone due to the risk ok irritation under the mechanical occlusion of masks. In contrast, Rudd et al. [16] have proposed retinoids alone. To minimize the risk of irritating dermatitis, Kaul et al. have suggested a short contact therapy, especially in those with pre-existing acne [41]. In this paper, it is also underlined that retinoids should be preferred to BP for their power in reducing acne flares. In our experience, the main results have been first obtained with daily skin care using appropriate products for acne. We feel also confident recommending zinc oxide formulations for reducing humidity, as did Teo et al. [20]. For moderate to severe forms, topical drugs have been necessary.

We have experienced a major a risk of irritation with products containing retinoids alone than with fixed combinations of retinoids and AB in hydrogel formulations. Excesses of erythema, dryness and desquamation have been observed with retinoids alone under the prolonged use of masks.

### 7.2. Systemic Treatment of Maskne

Systemic agents are summarized in Table 2 [31]. Oral antibiotics improve inflammatory lesions by inhibiting the growth of *C. acnes* within the pilosebaceous units. They are proposed for moderate to severe inflammatory acne and for all forms resistant to topical therapies. In order to avoid bacterial resistance, treatments are limited to continuous therapy for three to four months. Oral isotretinoin is the drug that combats the four pathogenetic aspects of acne: sebum production, follicular hyperkeratinization, inflammation, and *C. acnes*. It is well accepted as a treatment for severe, recalcitrant, and nodular acne. Oral contraceptives containing estrogen and progestin may reduce androgen action via a variety of mechanisms.

In the majority of articles about*maskne*, systemic therapies are not described in detail, while traditional acne treatments are suggested to obtain benefits [24,41,42]. Only the use of tetracycline is reported in the literature [2].

There are no reports supporting the use of isotretinoin or hormonal therapy for *maskne* so far.

Oral zinc may be taken into consideration as systemic drug alone or in support to oral AB [43]. Although the exact mechanism of action remains poorly elucidated, it is believed to act directly on the microbial inflammatory equilibrium and to facilitate antibiotic absorption when used in combination. Moreover, it seems to suppress sebum production by its anti-androgenic activity [44].

## 8. Conclusions

*Maskne* and all skin conditions related to the prolonged use of masks are emerging dermatoses. In this review, we proposed diagnostic criteria for mask-related acne and address the differential diagnoses of dermatological conditions triggered by mask-use. As far as therapeutic strategies, *maskne* requires the correct use of skin care cosmetics for acne prone skin and the use of topical treatments, i.e., sebum-regulators and emollients.

## Figures and Tables

**Table 1:** Topical therapies in acne.

Topical Agents	Effects	Type of Acne	Role in *Maskne* (Our Experience)
Benzoyl Peroxide (BP) 2.5–10% In monotherapy or combinations with topical antibiotics	Antibacterial No bacterial resistances	Mild papulopustular and mixed acne	Risk of irritation under mechanical occlusion
Topical antibiotics (AB) (e.g., Erythromycin 2% and Clindamycin 1%)	Antibacterial: high risk of bacterial resistances in monotherapy Anti-inflammatory	Mild papulopustular acne	Useful
Fixed combinations of BP and topical antibiotics	See above BP+ Reduction of bacterial resistance Enhances compliance	Mild-moderate papulopustular and mixed acne	
Topical retinoids (e.g., Tretinoin 0.025–0.1%, Adapalene 0.1–0.3%; Tazarotene 0.05–0.1%, Trifaroten 0.005%)	Comedolytic Anti-inflammatory Risk of dryness, peeling, erythema, and irritation	Comedonal acne	Risk of irritation under mechanical occlusion
Fixed combinations of retinoids and AB or PB	See above	Mild-moderate papulopustular and mixed acne	Hydrogel carrier formulations minimize local irritation [13]
Azelaic agent 20%	Mildly effective as: Comedolytic Antibacterial Anti-inflammatory Dyspigmentation	Mild comedonal acne Post-inflammatory dyspigmentation	No experience
Dapsone 5% gel	Unknown mechanism	Inflammatory acne, particularly in adult females with acne (poorly used)	
Salicylic acid 0.5–2%	Comedolytic	Mild comedonal acne	Risk of irritation

**Table 2:** Systemic therapies in acne.

Drugs		Dosage	Notes	*Maskne*
Antibiotics	Doxycycline	50 mg bid 100 mg/die or bid 40 mg modified release (mr)/die 20 mg mr bid	Not in children Not in pregnant Interaction with dairy products	Especiallyuseful for antinflammatory effect
	Minocycline	50 mg/die or bid 100 mg/die or bid		
	Tetracycline	250–500 mg bid		
	Azithromicyn	500 mg three times/week	Children Pregnant	
	Trimethoprim-sulfamethoxazole	160 mg/800 mg once to twice daily	Adults resistant to tetracycline/macrolides	No reports
Isotretinoin		0.5–1.0 mg/kg/die	Periodic monitoring of liver function tests, serum cholesterol, and triglycerides	
Hormonal agents	Oral contraceptives	According to the molecules	Only for women	
	Spironolactone	50–100 mg/die		

## Data Availability

Data sharing not applicable. No new data were created or analyzed in this study.

## References

[1] Giacalone S., Minuti A., Spigariolo C.B., Passoni E., Nazzaro G. (2021). Facial dermatoses in the general population due to wearing of personal protective masks during the COVID-19 pandemic: First observations after lockdown. Clin. Exp. Dermatol..

[2] Drozdowski R., Gronbeck C., Feng H. (2021). Mask-related acne in the COVID-19 pandemic: An analysis of Twitter posts and influencers. Clin. Exp. Dermatol..

[3] Yan Y., Chen H., Chen L., Cheng B., Diao P., Dong L., Gao X., Gu H., He L., Ji C. (2020). Consensus of Chinese experts on protection of skin and mucous membrane barrier for health-care workers fighting against coronavirus disease 2019. Dermatol. Ther..

[4] Altun E., Topaloglu Demir F. (2021). Occupational facial dermatoses related to mask use in healthcare professionals. J. Cosmet. Dermatol..

[5] Yaqoob S., Saleem A., Jarullah F.A., Asif A., Essar M.Y., Emad S. (2021). Association of Acne with Face Mask in Healthcare Workers amidst the COVID-19 Outbreak in Karachi, Pakistan. Clin. Cosmet. Investig. Dermatol..

[6] Techasatian L., Lebsing S., Uppala R., Thaowandee W., Chaiyarit J., Supakunpinyo C., Panombualert S., Mairiang D., Saengnipanthkul S., Wichajarn K. (2020). The Effects of the Face Mask on the Skin underneath: A Prospective Survey during the COVID-19 Pandemic. J. Prim. Care Community Health.

[7] Aravamuthan R., Arumugam S. (2020). Clinico-epidemiological study of mask induced acne due to increased mask use among health care workers during COVID pandemic in a tertiary care institute. Int. J. Res. Dermatol..

[8] Damiani G., Gironi L.C., Grada A., Kridin K., Finelli R., Buja A., Bragazzi N.L., Pigatto P.D.M., Savoia P. (2021). COVID-19 related masks increase severity of both acne (maskne) and rosacea (mask rosacea): Multi-center, real-life, telemedical, and observational prospective study. Dermatol. Ther..

[9] Montero-Vilchez T., Cuenca-Barrales C., Martinez-Lopez A., Molina-Leyva A., Arias-Santiago S. (2021). Skin adverse events related to personal protective equipment: A systematic review and meta-analysis. J. Eur. Acad. Dermatol. Venereol..

[10] Dinulos J.E., Dinulos J.G. (2021). Cutaneous coronavirus disease 2019 in children: A clinical primer for diagnosis and treatment. Curr. Opin. Pediatr..

[11] Szepietowski J.C., Matusiak Ł., Szepietowska M., Krajewski P.K., Białynicki-Birula R. (2020). Face Mask-induced Itch: A Self-questionnaire Study of 2,315 Responders during the COVID-19 Pandemic. Acta Derm. Venereol..

[12] Xerfan E.M.S., Facina A.S., Andersen M.L., Tufik S., Tomimori J. (2021). Acne flare-up due to mask wearing: A current pandemic scenario and its relationship with sleep. Skin Res. Technol..

[13] Foo C.C., Goon A.T., Leow Y.H., Goh C.L. (2006). Adverse skin reactions to personal protective equipment against severe acute respiratory syndrome—A descriptive study in Singapore. Contact Dermat..

[14] Hadžavdić A., Bukvić Mokos Z. (2021). Maskne: A New Entity in the COVID-19 Pandemic. Acta Dermatovenerol. Croat..

[15] Teo W.L. (2021). Diagnostic and management considerations for “maskne” in the era of COVID-19. J. Am. Acad. Dermatol..

[16] Rudd E., Walsh S. (2021). Mask related acne (“maskne”) and other facial dermatoses. BMJ.

[17] Özkesici Kurt B. (2021). The course of acne in healthcare workers during the COVID-19 pandemic and evaluation of possible risk factors. J. Cosmet. Dermatol..

[18] Luna P.C. (2020). Skin Microbiome as Years Go By. Am. J. Clin. Dermatol..

[19] Xu H., Li H. (2019). Acne, the Skin Microbiome, and Antibiotic Treatment. Am. J. Clin. Dermatol..

[20] Teo W.L. (2021). The “Maskne” microbiome-pathophysiology and therapeutics. Int. J. Dermatol..

[21] Dréno B., Dagnelie M.A., Khammari A., Corvec S. (2020). The Skin Microbiome: A New Actor in Inflammatory Acne. Am. J. Clin. Dermatol..

[22] Tucker S.B. (1983). Occupational tropical acne. Cutis.

[23] Sardana K., Sharma R.C., Sarkar R. (2002). Seasonal variation in acne vulgaris--myth or reality. J. Dermatol..

[24] Han C., Shi J., Chen Y., Zhang Z. (2020). Increased flare of acne caused by long-time mask wearing during COVID-19 pandemic among general population. Dermatol. Ther..

[25] Scarano A., Inchingolo F., Lorusso F. (2020). Facial Skin Temperature and Discomfort When Wearing Protective Face Masks: Thermal Infrared Imaging Evaluation and Hands Moving the Mask. Int. J. Environ. Res. Public Health.

[26] Brambilla L., Brena M., Tourlaki A. (2016). Textiles in dermatology: Our experience and literature review. G. Ital. Dermatol. Venereol..

[27] Nast A., Dréno B., Bettoli V., Mokos Z.B., Degitz K., Dressler C., Finlay A., Haedersdal M., Lambert J., Layton A. (2016). European evidence-based (S3) guideline for the treatment of acne-update 2016-short version. J. Eur. Acad. Dermatol. Venereol..

[28] Payne A. (2020). COVID-19: Skin damage with prolonged wear of FFP3 masks. BMJ.

[29] Abdali S., Yu J. (2021). Occupational Dermatoses Related to Personal Protective Equipment Used During the COVID-19 Pandemic. Dermatol. Clin..

[30] Singh G.K., Mitra B., Bhatnagar A., Mitra D., Talukdar K., Das P., Sandhu S., Sandhu S., Singh T. (2021). Unusual Spurts of Rosacea like Dermatoses, Posing a Diagnostic Dilemma during COVID-19 Pandemic: A Cross-Sectional, Observational Study from a Tertiary Care Centre. Indian J. Dermatol..

[31] Adalsteinsson J.A., Kaushik S., Muzumdar S., Guttman-Yassky E., Ungar J. (2020). An update on the microbiology, immunology and genetics of seborrheic dermatitis. Exp. Dermatol..

[32] Veraldi S., Angileri L., Barbareschi M. (2020). Seborrheic dermatitis and anti-COVID-19 masks. J. Cosmet. Dermatol..

[33] Tolaymat L., Hall M.R. (2021). Perioral Dermatitis. StatPearls. Treasure Island (FL).

[34] Donovan J., Kudla I., Holness L.D., Skotnicki-Grant S., Nethercott J.R. (2007). Skin reactions following use of N95 facial masks. Dermatitis.

[35] Liu H., Liu G. (2014). Definition, diagnosis and treatment of physical urticaria. J. Dermatol. Venereol..

[36] Zaenglein A.L., Pathy A.L., Schlosser B.J., Alikhan A., Baldwin H.E., Berson D.S., Bowe W.P., Graber E.M., Harper J.C., Kang S. (2016). Guidelines of care for the management of acne vulgaris [published correction appears in]. J. Am. Acad. Dermatol..

[37] Choi J.M., Lew V.K., Kimball A.B. (2006). A single-blinded, randomized, controlled clinical trial evaluating the effect of face washing on acne vulgaris. Pediatr. Dermatol..

[38] Zhao J., Wang Y., Jiang L., Mu Y.Z. (2020). The application of skin care product in acne treatment. Dermatol. Ther..

[39] Araviiskaia E., Dréno B. (2016). The role of topical dermocosmetics in acne vulgaris. J. Eur. Acad. Dermatol. Venereol..

[40] Gollnick H., Cunliffe W., Berson D., Dreno B., Finlay A.Y., Leyden J.J., Shalita A.R., Thiboutot D. (2003). Management of acne: A report from a Global Alliance to Improve Outcomes in Acne. J. Am. Acad. Dermatol..

[41] Kaul et Kaul S., Kaur I., Jakhar D. (2021). Facial Mask-related Acne and Acneiform Eruption During the Coronavirus Disease 2019 Pandemic: A Case Series. J. Clin. Aesthet. Dermatol..

[42] Searle T., Ali F.R., Al-Niaimi F. (2021). Identifying and addressing “Maskne” in clinical practice. Dermatol. Ther..

[43] Yee B.E., Richards P., Sui J.Y., Marsch A.F. (2020). Serum zinc levels and efficacy of zinc treatment in acne vulgaris: A systematic review and meta-analysis. Dermatol. Ther..

[44] Cervantes J., Eber A.E., Perper M., Nascimento V.M., Nouri K., Keri J.E. (2018). The role of zinc in the treatment of acne: A review of the literature. Dermatol. Ther..

